# Complete genome sequence of *Shinella* sp. strain 1A1, an ʟ-glucose-utilizing bacterium isolated from soil

**DOI:** 10.1128/mra.00217-25

**Published:** 2025-07-07

**Authors:** Yuki Doi, Akito Hama, Akira Nakamura

**Affiliations:** 1 Institute of Life and Environmental Sciences, University of Tsukuba, Tsukuba, Japan; 2 Microbiology Research Center for Sustainability (MiCS), University of Tsukuba 623473 , Tsukuba, Japan; 3 Tsukuba Institute for Advanced Research (TIAR), University of Tsukuba 13121, Tsukuba, Japan; University of Maryland School of Medicine, Baltimore, Maryland, USA

**Keywords:** ʟ-glucose utilization, *Shinella* sp., complete genome sequence

## Abstract

The complete genome sequence of an ʟ-glucose-utilizing bacterium, *Shinella* sp. strain 1A1 isolated from soil, was determined. Strain 1A1 contained a 3.45 Mb circular chromosome with 3,261 protein-coding genes, 9 rRNA, and 52 tRNA genes and 1.77 Mb and 44.5 kb circular plasmids with 1,623 and 51 protein-coding genes, respectively.

## ANNOUNCEMENT

**Fig 1:**
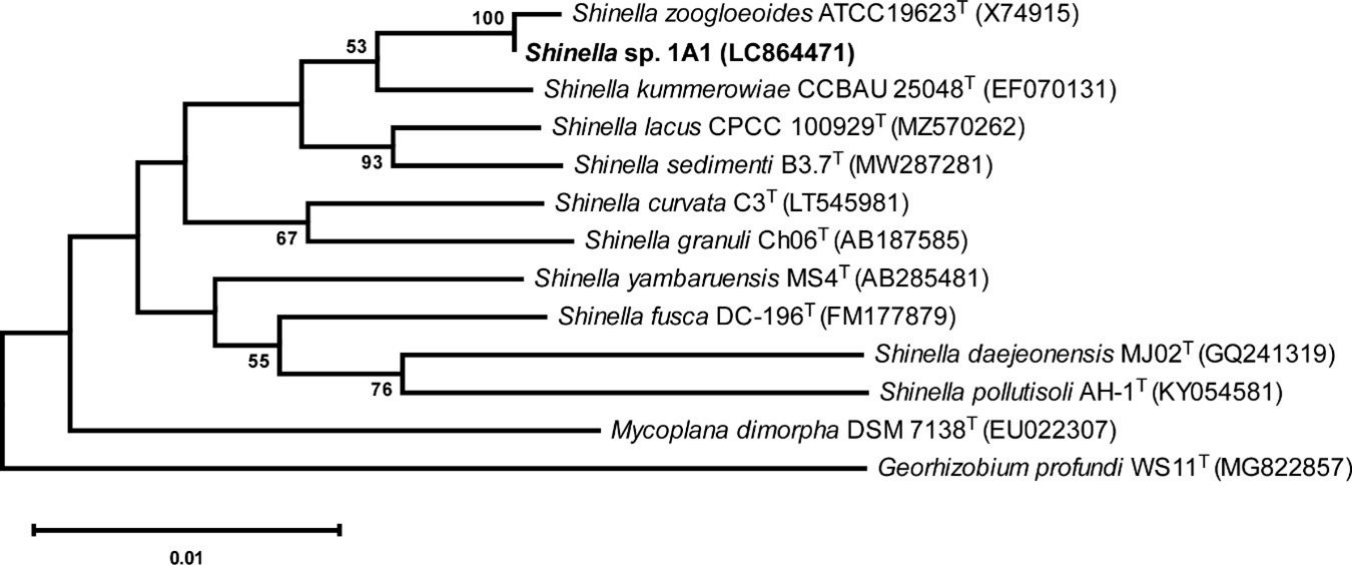
Phylogenetic tree based on the 16S rRNA gene sequence of strain 1A1 and related taxa. The tree was constructed by the neighbor-joining method in MEGA11. Accession numbers are shown in parentheses.

ʟ-glucose is an enantiomer of ᴅ-glucose, and living organisms were believed not to catabolize ʟ-glucose (1). Previously, we reported two ʟ-glucose-utilizing bacteria, belonging to the genera *Paracoccus* and *Luteolibacter* (2, 3). We isolated strain 1A1 as an ʟ-glucose-utilizer from a soil of the fertilizer test field in Tsukuba-Plant Innovation Research Center, University of Tsukuba (Tsukuba, Japan) by enrichment culturing in a minimal medium containing ʟ-glucose (ʟ-GlcMM, [2]) at 28°C with shaking, and colony formation on ʟ-GlcMM plates at 28°C for 4 days. The 16S rRNA gene sequence, determined with the colony-PCR fragment using primers 10F (5′-GAGTTTGATCCTGGCTCAG-3′) and 1492R (5′-GGTTACCTTGTTACGACTT-3′), and a phylogenetic analysis by the neighbor-joining method indicated that strain 1A1 is classified into the genus *Shinella*, with *S. zoogloeoides* as the closest related species (Fig. 1).

Strain 1A1 was cultured in ʟ-GlcMM for 72 h at 28°C. Genomic DNA extracted using Genomic-tip 20G (Qiagen) was purified using DNA Clean Beads (MGI Tech) and sheared to 10–25 kb with Megaruptor 3 (Diagenode) without size selection. The DNA library constructed using the SMRTbell Prep Kit 3.0 and SMRTbell gDNA Sample Amplification kit (PacBio) was sequenced with a Revio system and the Revio polymerase kit (PacBio). High-fidelity reads were generated by SMRT Link v. 13.1.0.221970 (PacBio). Trimming of PCR adapters and duplicate reads was conducted with lima v. 2.9.0 and pbmakdup v. 1.0.3 (PacBio), respectively. Short reads (≤1,000 bases) were eliminated using Filtlong v. 0.2.1 (https://github.com/rrwick/Filtlong), and the remaining 18,842 reads with an average length of 11,054 bp were assembled using Flye v. 2.9.3-b1797 (4) with default settings. Genomic circularity was confirmed by Bandage v. 0.8.1 (5), and the assembled data were confirmed using CheckM2 v. 1.0.1 (6) with 99.7% coverage and 1.58% contamination.

Strain 1A1 genomic DNA was a total of 5,264,811 bp with a GC content of 65.0% (*N*_50_ value, 3,446,576 bp), with a single circular chromosome (3,446,576 bp) and two circular plasmids, p1A1_1 and p1A1_2 (1,773,658 and 44,577 bp). Automatic annotation by Prokka v. 1.14.6 (7) indicated that the chromosome contained 3,261 protein-coding genes, 9 rRNA, and 52 tRNA genes. Plasmids p1A1_1 and p1A1_2 contained 1,623 and 51 protein-coding genes, respectively, with 5 tRNA genes in plasmid p1A1_1. This genome architecture resembles that of *S. zoogloeoides* strain Teo12 with the average nucleotide identity (ANI) value (8) of 97.63%. In contrast, the type strain of this species, ATCC 19623^T^, has a different architecture of 3.99 Mb chromosome and four plasmids of 373, 340, 109, and 34.7 kb, and showed ANI value of 88.46%, which is below the threshold of the species delineation (95%, 9). Most protein-coding genes in the chromosome and p1A1_1 of strain 1A1 showed high identities (>90%) to chromosome and plasmid p1_Teo12 of strain Teo12, respectively, except for some genes with less identities (<40%) located in several clusters. In contrast, those in p1A1_2 did not show any identities (<20%) to plasmids p2_Teo12 and p3_Teo12.

The complete genome sequence of strain 1A1 will support investigation on bacterial ʟ-glucose catabolism and contribute to the correct classification of the genus *Shinella*.

## Data Availability

The 16S rRNA gene sequence accession number for strain 1A1 is LC864471. The complete sequences of the chromosome, p1A1_1, and p1A1_2 of *Shinella* sp. strain 1A1 are available from DDBJ/EMBL/GenBank with accession numbers AP039671, AP039672, and AP039673, respectively. Raw sequence data were deposited in the DRA database under the accession number DRR656640 (BioProject number PRJDB20212 and BioSample number SAMD00884452).
